# Key Factors for Improving the Carcinogenic Risk Assessment of PAH Inhalation Exposure by Monte Carlo Simulation

**DOI:** 10.3390/ijerph182111106

**Published:** 2021-10-22

**Authors:** Ning Qin, Ayibota Tuerxunbieke, Qin Wang, Xing Chen, Rong Hou, Xiangyu Xu, Yunwei Liu, Dongqun Xu, Shu Tao, Xiaoli Duan

**Affiliations:** 1 School of Energy and Environmental Engineering, University of Science and Technology Beijing, Beijing 100083, China; qinning@ustb.edu.cn (N.Q.); aiyibuota@163.com (A.T.); yongmouren@163.com (X.C.); hourong0307@163.com (R.H.); jingling051@163.com (X.X.); 13294070361@163.com (Y.L.); 2 Chinese Center for Disease Control and Prevention, China CDC Key Laboratory of Environment and Population Health, National Institute of Environmental Health, Beijing 100021, China; wangqin@nieh.chinacdc.cn (Q.W.); xudq@chinacdc.cn (D.X.); 3 Laboratory for Earth Surface Processes, College of Urban and Environmental Sciences, Peking University, Beijing 100871, China; taos@pku.edu.cn

**Keywords:** risk assessment, Monte Carlo simulation, PAHs, exposure parameter, sensitivity analysis

## Abstract

Monte Carlo simulation (MCS) is a computational technique widely used in exposure and risk assessment. However, the result of traditional health risk assessment based on the MCS method has always been questioned due to the uncertainty introduced in parameter estimation and the difficulty in result validation. Herein, data from a large-scale investigation of individual polycyclic aromatic hydrocarbon (PAH) exposure was used to explore the key factors for improving the MCS method. Research participants were selected using a statistical sampling method in a typical PAH polluted city. Atmospheric PAH concentrations from 25 sampling sites in the area were detected by GC-MS and exposure parameters of participants were collected by field measurement. The incremental lifetime cancer risk (ILCR) of participants was calculated based on the measured data and considered to be the actual carcinogenic risk of the population. Predicted risks were evaluated by traditional assessment method based on MCS and three improved models including concentration-adjusted, age-stratified, and correlated-parameter-adjusted Monte Carlo methods. The goodness of fit of the models was evaluated quantitatively by comparing with the actual risk. The results showed that the average risk derived by traditional and age-stratified Monte Carlo simulation was 2.6 times higher, and the standard deviation was 3.7 times higher than the actual values. In contrast, the predicted risks of concentration- and correlated-parameter-adjusted models were in good agreement with the actual ILCR. The results of the comparison suggested that accurate simulation of exposure concentration and adjustment of correlated parameters could greatly improve the MCS. The research also reveals that the social factors related to exposure and potential relationship between variables are important issues affecting risk assessment, which require full consideration in assessment and further study in future research.

## 1. Introduction

Health risk is defined as the likelihood of an adverse effect in the population due to exposure to a contaminant. Accurate assessment of the health risk is of great significance for the determination of adverse effects and disease prevention. Monte Carlo simulation (MCS) is a computational technique used in the health risk assessment (HRA) [1,2,3]. Compared to point estimation, probabilistic risk estimation based on MCS allows considerably greater accuracy in describing the variables’ uncertainty and improves the understanding of contaminants’ environmental behaviors [4,5]. Thus, it makes the assessment more informative to risk managers [6,7]. MCS is widely used in the risk estimation of multimedia exposure and parameter sensitivity qualification [8,9,10]. Recently, MCS has been combined with the physiologically based pharmacokinetic model to determine the relationship between internal exposure dose and cancer risk [11]. Improved, two-dimensional MCS has also been used to directly provide specific guidance for the risk control of pollutant exposure [12,13]. With the development of exposure parameter research, such as the publication of Exposure Factor Handbook of U.S. EPA and Exposure Factors Handbook of Chinese Population, sufficient exposure parameters have been provided to support use of the MCS method in exposure estimation. Therefore, MCS has gradually replaced point estimation and is widely applied in the risk assessment of multiple exposure routes [14,15,16].

The traditional assessment model based on the MCS approach also suffers from several drawbacks. First, MCS treats input variables as random variables with known or estimated probability density functions. The distribution model’s selection of parameters is obviously affected by the subjectivity of the researcher. It is difficult to determine how well the model represents the actual conditions. Second, correlated variables are treated independently in traditional risk assessment, which may increase the estimation uncertainty. Mathematical relationships exist among some variables. For example, positive correlation can be detected between a subject’s water intake rate and body weight. It has been reported that a correlation with an absolute value of the coefficient greater than 0.6 will have larger effects on the output distributions [5]. In a traditional MCS risk assessment model, however, these variables are often treated as independent variables. This treatment will inevitably result in increasing uncertainty. Finally, the results of risk assessment based on MCS are difficult to validate. Risk estimation based on MCS usually focuses on a large population group. It is difficult to obtain the actual risk of each participant in the population by sampling due to the high cost.

Here, we wanted to use data from a large-scale investigation of individual exposure to polycyclic aromatic hydrocarbons (PAHs) to explore the key factors for improving the accuracy and decreasing the uncertainty of assessment based on MCS. PAHs are semi-volatile organic compounds that are generally formed by the incomplete combustion of fossil fuels and biomass fuels [17,18,19]. They are associated with potentially toxic, mutagenic, and carcinogenic effects [20,21]. In accordance with the currently available epidemiological and toxicology data, exposure to high levels of PAHs has been proven to be a potential cause of skin, lung, bladder, and gastrointestinal cancers [22,23,24,25,26]. Due to the wide range of sources, strong carcinogenicity, and large emissions of PAHs, health risks caused by them have been a topic of public concern [27,28,29]. Actual carcinogenic risk though PAH inhalation was estimated by measured PAH concentration and population exposure parameters. Traditional assessment model based on MCS and improved models from three following aspects were also applied to evaluate the carcinogenic risk though PAH inhalation. (1) PAH exposure concentration was adjusted by the weight of population density in the research area, (2) the target population was age-stratified to increase the accuracy of exposure parameters, and (3) correlated parameters were adjusted by a regression model. The goodness of fit of the models was evaluated quantitatively by comparison with the actual risk of 2740 participants. The purpose of this study was to screen the key factors affecting the MCS method and find methods to improve the assessment based on MCS.

## 2. Materials and Methods

### 2.1. Research Area and Participants

**Figure 1:**
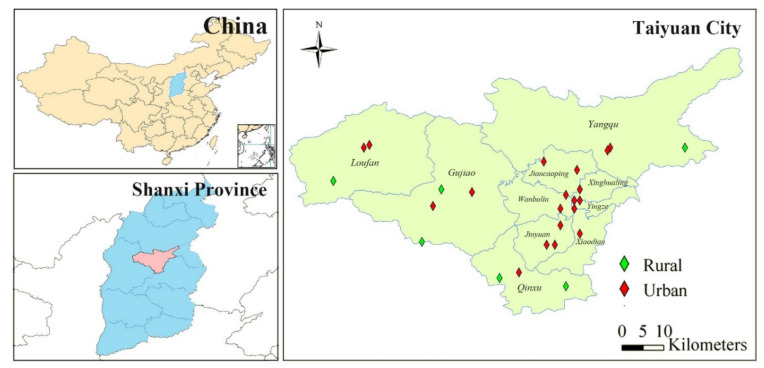
Atmosphere sampling sites in Taiyuan City. Six districts include Jiancaoping (JCP), Xinghualing (XHL), Wanbolin (WBL), Jinyuan (JY), Xiaodian (XD), and Yingze (YZ); four counties include Yangqu (YQ), Loufan (LF), Gujiao (GJ), and Qingxu (QX).

Taiyuan City, the capital of Shanxi Province, was selected as the research area in this study (Figure 1). Taiyuan, as an important industrial base in northern China, has long been plagued by air pollution, especially PAH pollution, due to high energy consumption and poor air diffusivity [30,31,32,33]. According to statistics, the annual consumption of oil in Taiyuan was 1.9 million tons standard coal equivalent, and the annual coal consumption was 79.0 million tons of coal, accounting for 20.4% of the total oil consumption and 29.4% of the coal consumption in Shanxi Province, respectively [34]. In this study, a total of 25 sampling sites were selected and covered all the six districts and four counties of Taiyuan City. Because the exposure scenarios of urban and rural areas were different, all the 25 sampling sites were classified into urban and rural groups, respectively. The population nearby was also classified accordingly.

To obtain the exposure parameters including the height and body weight of the population in Taiyuan, field measurements were conducted among adult residents according to a designed statistical sampling method. The field measurement covered a sample size of 2740 local adult residents. The basic information of the participants is shown in Table 1. Age structure and sex ratio are two important factors to determine the representativeness of the participants. These two factors of participants in Taiyuan were compared with the census data of Taiyuan (Figure S1). Similar age structure and sex ratio can be found in subjects from most age groups and the target population [35]. It can be considered that the sample size is sufficient to reflect the characteristics of the population in Taiyuan.

### 2.2. Sampling and Analytical Methods

Atmospheric particulate and gaseous-phase PAH samples were collected from 25 sampling sites in each region using passive air samplers with a PUF disk and glass fiber filter (Figure 1). Atmospheric samples were collected in three periods of a year including spring, summer–autumn, and winter between 2009 and 2010. After sampling, the PAHs in the gas and particulate phase samples were Soxhlet extracted, purified by alumina silica gel column, and measured using an Agilent 6890 N gas chromatograph coupled with an Agilent 5975 mass spectrometer with an electron impact ion source. Details of the analytical and quantification procedures have been described previously [36,37]. Fifteen priority controlled PAHs were identified, including acenaphthene (Ace), acenaphthylene (Acy), fluorine (Flo), phenanthrene (Phe), anthracene (Ant), fluoranthene (Fla), pyrene (Pyr), benz(a)anthracene (BaA), chrysene (Chr), benzo(b)fluoranthene (BbF), benzo(k)fluoranthene (BkF), benzo(a)pyrene (BaP), dibenz(a, h) anthracene (DahA), indeno(1,2,3-cd)pyrene (IcdP), and benzo(ghi) perylene (BghiP). The annual average PAH concentration was calculated and used to represent the PAH exposure level of the population.

### 2.3. Risk Characterization

Based on the USEPA risk assessment protocol [38], the carcinogenic risk was characterized by the incremental lifetime cancer risk (ILCR). The ILCR of the studied population was estimated based on the following equation:

(1) $$\mathrm{ILCR}=\frac{{\mathrm{BaP}}_{\mathrm{eq}}\times \mathrm{IR}\times \mathrm{EF}\times \mathrm{ED}\times {\mathrm{CSF}}_{\mathrm{BaP}}}{\mathrm{BW}\times \mathrm{AT}}$$

(2) $${\mathrm{BaP}}_{\mathrm{eq}}=\sum C_{i}\times {\mathrm{TEF}}_{i}$$

where BaP_eq_ is the BaP equivalent concentration (BaP_eq_) in gaseous phase and particulate phase (ng·m^−3^), IR is the inhalation rate (m^3^·day^−1^), EF is the exposure frequency (365 day·year^−1^), ED is exposure duration (53 year), BW is the body weight (kg), and AT is the average time (70 years × 365 day·year^−1^). CSF_BaP_ is the inhalation cancer slope factor for BaP (3.14 per mg·kg^−1^·day^−1^).

The BaP_eq_ and the toxicity equivalency factors (TEFs) were used to express the carcinogenic risk of PAH mixtures. The BaP_eq_ based on the BaP toxicity was incorporated using Equation (2), where C_i_ is the concentration of the PAH species in atmospheric samples, and TEF_i_ is the toxic equivalence factor of the congener of PAH, i. Table S1 lists the PAHs and TEFs used in the calculation associated with the evidence of cancer in PAH-exposed individuals.

Parameters in calculating individual risk are different from those used in Monte Carlo simulation. For the risk assessment of participants, the monitored PAH concentration in the closest sampling site to the participant was taken as the individual exposure level. Body weight was obtained directly by field measurement. IR was estimated by the basal metabolic rate method based on body weight and height. The details of estimation are provided in Table S2 and Text S1. For the Monte Carlo simulation, the exposure parameters of adults in Taiyuan were collected from the Exposure Handbook of Chinese Population.

### 2.4. Monte Carlo Simulation

Monte Carlo simulations were used to generates samples from probability distributions describing the range of results and the sensitivities of relative parameters. Here, three parameters including BaP_eq_ concentration, body weight, and inhalation rate were considered as variables. Log-normal distributions were applied to describe variables, because log-normal is the most widely applied model in studies on the exposure parameters [39,40]. Distribution of concentration was obtained by Gaussian fitting of the monitoring data from 25 sampling sites. BW and IR were derived based on the exposure parameter collected from the Exposure Factors Handbook of Chinese Population [41].

Once the distribution of the variables was fixed, the computer selected a value for each variable at random from the specified distribution and calculated the corresponding risk. This process was repeated many times, the set of input values and corresponding estimate of risk were saved each time. In an MCS process, each calculation is referred to as an iteration, and a set of iterations is called a simulation. The Monte Carlo simulation program was written in Python 3.7. Iterations ranging from 5000 to 50,000 were performed to test convergence and the stability of the numerical output. As a result, a total of 30,000 iterations were sufficient to ensure a subgroup with the least population (age 60–70) can obtain convergence results. Rank correlation coefficients between each input variable and the carcinogenic risk were calculated by Spearman correlation analysis. The sensitivity of each input variable was quantified by comparing the rank correlation coefficients [42].

## 3. Results and Discussion

### 3.1. Individual Actual Risks Based on Measured Parameters

The carcinogenic risk of 2740 participants was assessed based on the PAH concentrations and parameters measured. We assumed that the subjects could represent the population of Taiyuan; thus, the calculated risk value based on the actual measurement was close to the real risk of the population. Therefore, in our research, risks based on measured data were considered as actual risk values to evaluate the goodness of fit of the Monte Carlo simulations.

The cancer risk of the whole population varied from 7.53 × 10^−7^ to 4.67 × 10^−5^, with a median of 4.83 × 10^−6^. Compared with risks reported from the other cities in China, ILCR in Taiyuan was much higher than that reported in Xingtai, Hebei, with an average ILCR of 3.4 × 10^−7^ [43]. However, ILCR in Taiyuan was lower than that of people in some typical industrial cities such as Nanchong, Sichuan Province, with a reported value of 1.2 × 10^−5^ [44] and Changzhou with a value of 3.6 × 10^−3^ [45].

**Figure 2:**
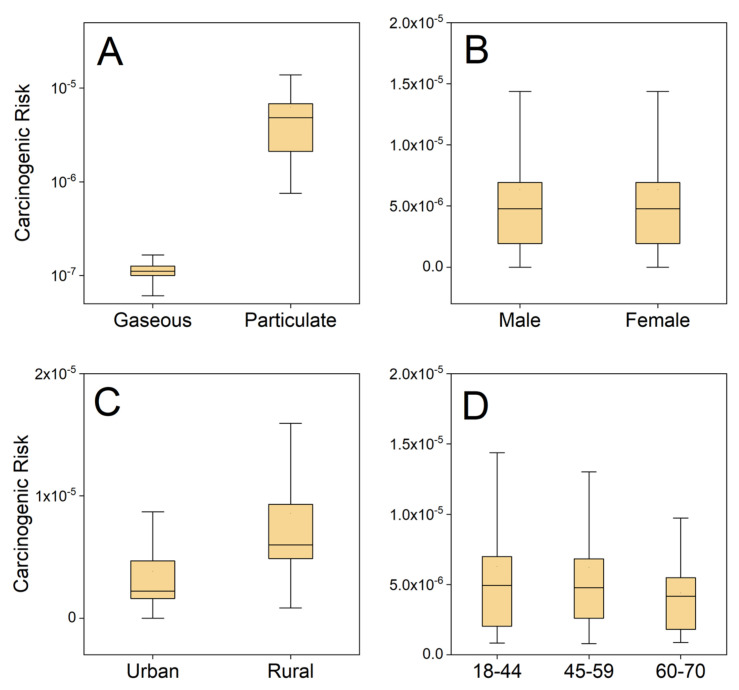
Influencing factors of ILCR of participants from Taiyuan. Difference of risk (**A**) between exposure phrases, (**B**) between genders, (**C**) between urban and rural populations, and (**D**) among age subgroups.

Influencing factors of both environmental concentration and population exposure characteristics were studied (Figure 2). Exposure assessment indicated that local residents had a mean gaseous exposure level of 16.28 ng kg^−1^ day^−1^, which was in the same order of magnitude as the level of particulate PAH_15_ with a mean value of 21.13 ng kg^−1^ day^−1^. However, it can be seen from Figure 2A that particulate PAHs had a significantly higher BaP_eq_ contribution and higher risk than did gaseous phase (Kruskal-Wallis test, *p* < 0.01). Previous research suggested PAHs with higher molecular weight were also more carcinogenic and more easily absorbed onto particles. Our findings are consistent with previous results [46].

In addition to the influence of environmental concentration, the exposure behaviors and physiological parameters of different subgroups of the population are often different, resulting in variation in the health risk. Kruskal-Wallis analysis was used to compare people of different genders, residential areas, and age groups. Although a significant difference can be detected between the body weight and inhalation rate of male and female participants (Kruskal-Wallis, *p* < 0.01), no significant carcinogenic risk difference was found between genders (*p* = 0.11). Similarly, no significant risk difference was observed among age groups (*p* = 0.64). These results also indicated that the influence of physiological parameters on exposure risk was not as obvious as we expected. In contrast, the health risk of rural participants (geomean, 5.9 × 10^−6^) was significantly higher than that of urban ones (geomean, 3.4 × 10^−6^). Based on the results above, it can be inferred that different exposure levels in urban and rural areas may be more important factors than physiological parameters.

According to the criteria suggested by the U.S. EPA, a one-in-a-million chance of an additional human cancer over a 70 year lifetime is an acceptable level of risk, and a one-in-ten-thousand or greater chance is considered to be serious risk. Based on this, almost the entire population within the research area exceeded acceptable limits. However, none of the participant’s risk exceeded the serious level. The results suggested local people had low probability of potential cancer risk. 

### 3.2. Population Risk Based on Monte Carlo Simulation

#### 3.2.1. Parameter Distribution Estimation

According to the results of the Shapiro-Wilk test (*p* > 0.05), the BaP_eq_ concentration can be described by a log-normal distribution LN(2.21, 1.01). After ranking these data from least to greatest, plotting positions (proportions) for use in a cumulative probability distribution were calculated as:

Proportion = rank / (number of samples + 1)(3)

**Figure 3:**
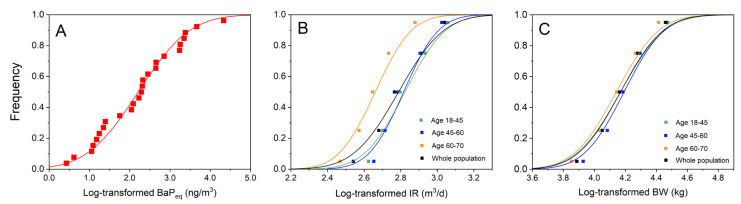
Fitting of exposure parameters in the risk assessment (**A**) BaP_eq_ concentration, (**B**) inhalation rate of populations from different age groups, and (**C**) body weight of populations from different age groups.

Distribution of concentration was obtained by Gaussian fitting of the monitoring data from 25 sampling sites (Figure 3A, *R*^2^ = 0.941). In a traditional assessment model, it is difficult to collect the physiological parameters of participants due to the large population and high cost. The development of research on exposure parameters in recent years has provided great convenience to the accurate assessment of health risk through the Monte Carlo method. We obtained quartiles and medians from the Exposure Factors Handbook of Chinese Population. The weight and IR area taken as *x* values and the corresponding quartiles as *y* values. The weight and IR distribution of the population were also fitted using a cumulative distribution function of the log-normal model (Figure 3B,C). The fitting parameters describing the curves are shown in Table 2.

#### 3.2.2. Comparison of Risk Distribution between the Two Methods

**Figure 4:**
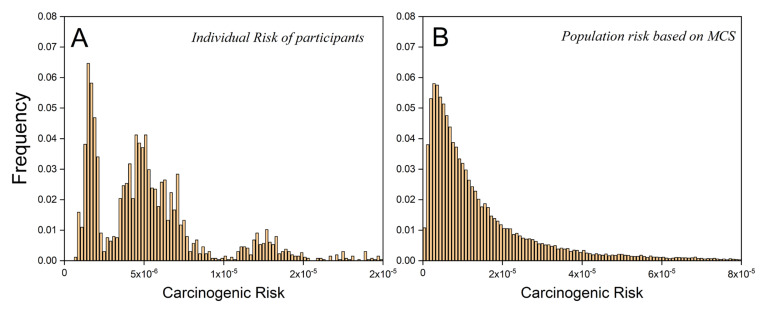
Risk distribution obtained by (**A**) individual assessment and (**B**) traditional assessment model based on MCS.

The risk distribution of individual participants and the result derived by Monte Carlo simulation are illustrated in Figure 4A,B, respectively. Obvious difference can be observed between the distribution mode of two methods. Three peaks can be found in the actual risk distribution of 2740 participants. The three peaks contained a population of 741, 1594, and 405, respectively. The age composition of three peaks was analyzed. No obvious age difference was observed among three groups. If the population was divided into age subgroups by every 10 years, people in age group 30–40 had the highest proportion in three peaks with percentages of 25.9%, 26.0%, and 32.1%, respectively. In contrast, the analysis of the spatial variation of risk found that a total of 51.2% of the population in the first peak was from Loufan District, which was considered to be a relatively low-pollution area in Taiyuan. For the third peak with high risk, about 49.5% of the population was from Jiancaoping District, which is a highly polluted area in the center of Taiyuan City. The results showed that a large proportion of people at high risk were from the same region. The same applied to the people at low risk. This showed that spatial variation of PAHs had an obvious impact on risk distribution. Meanwhile, population density may also be an important reason for this result. In summary, the statistical analysis showed that the arithmetic mean of actual ILCR was 6.25 × 10^−6^, with a standard deviation of 6.40 × 10^−6^. The average risk according to the Monte Carlo simulation was 1.63 × 10^−5^ with a standard deviation of 2.37 × 10^−5^.

#### 3.2.3. Parameter Sensitivity Analysis

Comparing the Monte Carlo results with the actual risk calculated based on 2740 subjects, it can be found that Monte Carlo simulation overestimated the risk level of the population, and the range of uncertainty introduced was much larger than that of the real population. A detailed sensitivity analysis was conducted to identify the input variables that were critical to the accuracy of the risk assessment.

**Figure 5:**
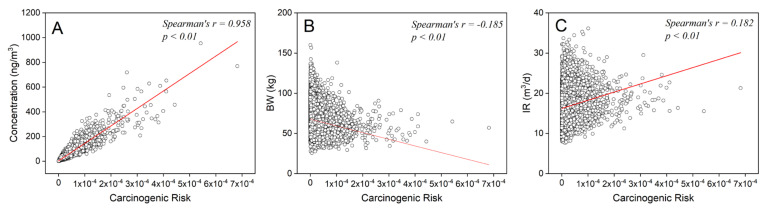
Parameter sensitivity analysis: (**A**) concentration, (**B**) body weight, and (**C**) intake rate.

In each iteration, the set of input variables and the corresponding risk were saved. The sensitivity of each input variable was quantified by comparing the Spearman rank correlation coefficients (Figure 5). The greater the coefficient between parameter and risk, the more sensitive the parameter is. The result showed that BaP_eq_ concentration had the highest parameter sensitivity with Spearman’s *r* = 0.958. This is consistent with the previous research [47,48]. Exposure concentration was a more sensitive factor than exposure parameters in affecting the accuracy of assessment in this research. The other two parameters, BW and IR, had comparable sensitivity, with *r* values of −0.185 and 0.182.

### 3.3. Key Factors for Improving the Monte Carlo Simulation

The comparison showed that differences exist between the risk calculated by the traditional model and the actual risk. Combined with the results of parameter sensitivity, the process of exposure parameter estimation can be an important source of uncertainty. Three possible causes of the high uncertainty existed in the process of parameter estimation. First, when fitting the concentration distribution, all the 25 samples had the same weight, which ignored the spatial variation of pollutants and population density. As a result, the importance of sample sites in densely populated areas was reduced, and the importance of concentration from some sparsely populated areas was overestimated. Second, the physiological parameters of the population differed greatly, which brought great uncertainty when they were included in the same group. Third, obvious positive correlation existed between BW and IR (Figure S2), but the two variables were randomly sampled in the traditional Monte Carlo process separately. We could not find the relationship between randomly sampled BW and IR (Figure S3).

Correspondingly, we revised the traditional model from three aspects to solve the problems above and reduce the uncertainty caused by parameter estimation. First, a new BaP_eq_ concentration distribution curve was established based on the BaP_eq_ exposure level of individual participants instead of sampling sites. Thus, the weight of each sample point in the fitting model would be reflected through the number of people near the sites. Second, stratified analysis was used in the population to reduce the difference of physiological parameters in each subgroup. According to the characteristics of exposure parameters, the population was divided into three age groups: 18–44, 45–59, and 60–70 years old. Surrogate samples were generated by MCS. The number of surrogate samples in the age group was determined by the proportion of the age group in the whole population. Finally, the regression relationship between IR and BW was established. IR was replaced by an equation with BW to calculate the carcinogenic risk (Equation (4)). Only the concentration and body weight were used as variables for Monte Carlo simulation. The purpose of the three adjustments was to explore the influence of parameters on the simulation results, increase the accuracy, and reduce the uncertainty of MCS.

(4) logIR=0.679logBW−0.281

### 3.4. Improved Models

#### 3.4.1. Concentration-Adjusted Monte Carlo

**Figure 6:**
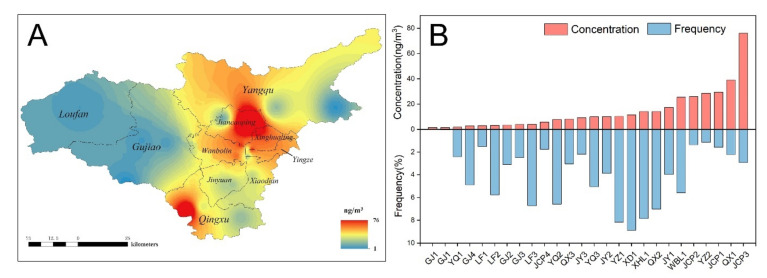
(**A**) Spatial distribution of BaP_eq_ concentration in Taiyuan City and (**B**) participant frequency of each sampling site.

Different sampling points had different contributions to the population ILCR. The weight of each sampling site depended on the population density near the sites. The spatial distribution of BaP_eq_ concentration in Taiyuan were constructed by ArcGIS software using the inverse distance weighting method (Figure 6A). The participant frequency of each sampling site is illustrated in Figure 6B. From the figure, we can clearly see that the pollution level in the urban area was higher than that in the suburbs. The highest pollution level was found in the north of the city. Jiancaoping District had the highest concentration. Site JCP3 had the greatest BaP_eq_ concentration of 76.09 ng·m^−3^. Fortunately, the participant frequency of four sampling sites in Jiancaoping was relatively low. The residence of only 80 participants was near the sampling site JCP3. The highest frequency was at a sampling site in Xiaodian District, followed by Yingze and Xinghualing.

In order to take the factor of population density into account, we assumed that the population density around the sampling sites can be reflected by the frequency of subjects. Thus, the BaP_eq_ concentrations from each sampling site were replaced by exposure levels of individual participants nearby to build a new BaP_eq_ concentration distribution. The BaP_eq_ was also fitted using log-normal distribution LN(2.419,1.317). The risk of the population was estimated by another Monte Carlo simulation. The arithmetic mean of participants was 8.23 × 10^−6^, with a standard deviation of 8.48 × 10^−6^.

#### 3.4.2. Age-Stratified Monte Carlo

In addition to the influence of exposure concentration, uncertainty existed in the physiological parameters of participants also. Collected data showed that there always were big gaps between the exposure parameters of different life stages. The average inhalation rate of people in age of 18~44 years was 18% higher than that of people aged from 60~70 years old. Dividing the population into subgroups is a commonly used method to reduce the uncertainty. We categorized people potentially being exposed to PAHs in Taiyuan into three subgroups based on their life stages and exposure parameters: 18–44, 45–59, and 60–70 years old. Three levels of risk assessment were evaluated by Monte Carlo simulation. The iterations of Monte Carlo simulation were decided on the local population structure. The iterations of age groups 18–44, 45–59, and 60–70 years were 19,379, 8172, and 2449 in accordance with statistics of the Shanxi 2010 census. The surrogate samples of three levels of Monte Carlo simulation were analyzed. As a result of the stratified Monte Carlo, the arithmetic mean of participants was 1.6 × 10^−5^, with a standard deviation of 2.4 × 10^−5^.

#### 3.4.3. Parameter-Correlation-Adjusted Monte Carlo

Inhalation rate was replaced by the relationship between IR and BW in (Equation (4)). The concentration and weight were used as variables and randomly generated by MCS. As a result of the parameter-correlation-adjusted Monte Carlo, the arithmetic mean of ILCR was 8.10 × 10^−6^, with a standard deviation of 1.26 × 10^−5^.

### 3.5. Comparison of Improved Models

**Figure 7:**
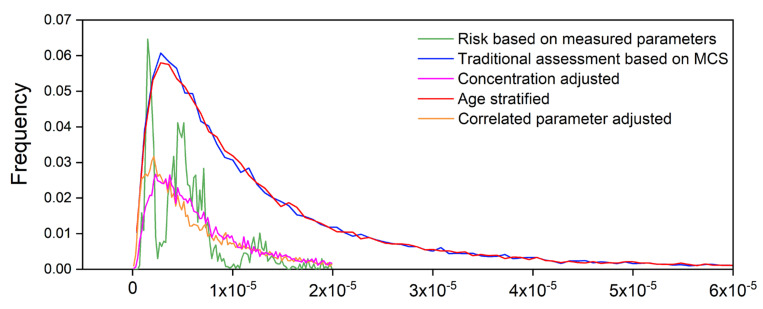
Risk distributions of five assessment models.

The risk distributions of four Monte Carlo simulations were compared with the actual risk as shown in Figure 7. It can be seen that the actual cancer risk had an irregular distribution. Therefore, it is difficult to accurately reflect the real distribution of risk using Monte Carlo simulation. However, we can evaluate the goodness of fit of the simulation based on two indicators including the accuracy and the variation. Simulation accuracy was quantitatively characterized by comparing the arithmetic mean and median values. The variation was evaluated by comparing the standard variation and the range. Risk distribution curves and statistics showed that no difference was found between the traditional model and the age-stratified model. The mean and median values of MCS surrogates was 2.6 and 1.9 times higher than the actual risk based on measured parameters. The standard deviation was 3.7 times higher, and the range was 1.5 orders of magnitude larger. The result indicated there was a big gap between the ILCR predicted by traditional and age-stratified MCS and the actual ILCR, and the age-stratification method did not improve the accuracy of prediction in this study. There maybe two reasons accounting for the failure of age-stratified MCS. First, according to the results of sensitivity analysis, the Spearman rank correlation coefficients between these two parameters and ILCR were 0.19 and 0.18. BW and IR were not the most sensitive parameters in this study. The effect of age stratification on reducing uncertainty is relatively small. Second, our target group was adults in Taiyuan, and the proportion of adolescents and the elderly in the population was relatively low, which means that the difference of physiological parameters was not obvious. Therefore, the difference between the two simulation methods in Taiyuan was not obvious.

In contrast, much better prediction results can be seen in Figure 7 by adjustment of the concentration and parameter correlation. The arithmetic mean and median of the concentration-adjusted model were 32% and 18% higher than the actual ILCR. The arithmetic mean and median of the correlated-parameter-adjusted model were only 29.5% and 2% higher than actual risk, respectively. Elimination of parameter-related effects greatly increased the accuracy of prediction. The predicted median value almost coincided with the actual risk. Furthermore, the overlap between the curves of the two models and the green curve representing the actual risk showed a great reduction in the variation of these two models. The standard deviation of the concentration-adjusted and parameter-correlation-adjusted models was 1.97 times higher than the actual ILCR. Concentration adjustment had a better effect in reducing the standard deviation.

To provide a reasonable and effective method to improve the MCS method, the cost of each improvement also needs to be considered. In this study, the adjustment of concentration requires a better understanding of population spatial distribution and density. The acquisition of this information always requires a lot of additional work. If we want more accurate assessment results, it is a good choice to replace the site sampling with individual sampling, which requires even more resources. In contrast, the cost of parameter correlated adjustment is relatively low. Due to the development of exposure science, the relationship between parameters has been well documented in different races, ages, and genders. Correlated parameter adjustment can effectively improve the accuracy at low cost. Age stratification requires more detailed physiological parameter research of different age groups. It is also a low-cost method for countries and regions with relatively perfect parameter research. In addition, it should be noted that although the effect of age stratification was not obvious in this study, it is still possible to greatly improve the accuracy of prediction for populations with a more complex age structure.

## 4. Conclusions

In recent years, risk assessment has become an area of public concern. Due to the complexity of individual exposure environment and high cost of monitoring, risk assessment needs to find a balance between cost and accuracy. Therefore, the improvement of risk assessment based on MCS not only has important scientific value but also carries positive socioeconomic impacts. In this research, the health risk of the target population was evaluated through the traditional assessment method and also through three improved models. The results were compared with risk estimated based on the measured parameters of 2740 participants. The comparison suggested that the adjustment of concentration and correlated parameters can greatly improve the accuracy and reduce the uncertainty of risk assessment. In contrast, for people with small physiological differences, stratified analysis cannot improve the model effectively. The findings of this study are significant as they can help improve the accuracy of the risk assessment. Moreover, the result suggested that in addition to laboratory experiments and field measurements, more attention should be paid to collecting the sociological information of populations and finding out potential relationship between exposure parameters. Full consideration of these factors can greatly improve the prediction of health risk, reduce the scale of sampling, and minimize the cost of experiments.

## Figures and Tables

**Table 1:** Basic information of participants in Taiyuan.

	N	Height (cm)		Body Weight (kg)		Inhalation Rate ^a^ (L/min)	
		Mean	SD	Mean	SD	Mean	SD
SEX							
Male	1305	171	5.41	67.7	8.8	14.04	0.73
Female	1435	160	5.80	56.9	10.1	11.07	1.17
AREA							
Urban	1382	166	7.53	63.0	11.0	12.51	1.83
Rural	1358	164	7.84	60.6	10.6	12.30	1.71
Age group							
16 ~< 25 years	450	166	8.06	57.4	10.7	12.17	2.03
26 ~< 35 years	626	166	7.48	61.3	10.8	12.48	1.87
36 ~< 45 years	737	164	7.34	62.9	10.5	12.47	1.67
46 ~< 55 years	618	165	7.67	63.7	10.8	12.56	1.64
56 ~< 65 years	303	163	8.31	63.0	11.0	12.14	1.66
66 ~< 75 years	6	171	10.89	70.5	12.0	11.68	1.60

^a^ Inhalation rates were estimated based on the basal metabolic rate (BMR), the estimation method is provided in Supplementary Materials, Text S1.

**Table 2:** Distribution of exposure parameters for Monte Carlo simulation.

Age Subgroup (Years)	BW (kg)			IR (m^3^·day^−1^)		
	*a*	*b*	*R* ^2^	*a*	*b*	*R* ^2^
18–44	4.169	0.225	0.991	2.823	0.175	0.944
45–59	4.192	0.204	0.991	2.816	0.150	0.986
60–70	4.142	0.216	0.993	2.660	0.157	0.988
Whole population	4.169	0.218	0.992	2.788	0.195	0.995

Exposure parameters considered as random variables follow a log-normal distribution, LN(*a*, *b*), for different age groups. Parameters *a* and *b* are the average value and standard deviation of log-transformed parameters, respectively.

## Data Availability

Not applicable.

## Supplementary Materials

The following are available online at https://www.mdpi.com/article/10.3390/ijerph182111106/s1, Figure S1: Comparison of age structure difference between participants and local population, Figure S2: Correlation between IR and BW in participants, Figure S3: Relationship between inhalation rate and body weight by traditional Monte Carlo simulation, Table S1: Toxicity equivalency factors (TEFs) and reference dose for the PAHs [49], Table S2: Estimation of BMR (basal metabolic rate) of population in Taiyuan, Text S1: Estimation of inhalation rate [50,51].

## References

[1] Yang Y., Xu X., Georgopoulos P.G. (2009). A Bayesian population PBPK model for multiroute chloroform exposure. J. Expo. Sci. Environ. Epidemiol..

[2] Lester R.R., Green L.C., Linkov I. (2007). Site-specific applications of probabilistic health risk assessment: Review of the literature since 2000. Risk Anal..

[3] Glorennec P., Bemrah N., Tard A., Robin A., Le Bot B., Bard D. (2007). Probabilistic modeling of young children’s overall lead exposure in France: Integrated approach for various exposure media. Environ. Int..

[4] Mishra H., Singh J., Karmakar S., Kumar R. (2021). An integrated approach for modeling uncertainty in human health risk assessment. Environ. Sci. Pollut. Res..

[5] Burmaster D.E., Anderson P.D. (1994). Principles of Good Practice for the Use of Monte Carlo Techniques in Human Health and Ecological Risk Assessments. Risk Anal..

[6] Thompson K.M., Burmaster D.E., Crouch E.A. (1992). Monte Carlo Techniques for Quantitative Uncertainty Analysis in Public Health Risk Assessments. Risk Anal..

[7] Dong Z., Liu Y., Duan L., Bekele D., Naidu R. (2015). Uncertainties in human health risk assessment of environmental contaminants: A review and perspective. Environ. Int..

[8] Huang J., Wu Y., Sun J., Li X., Geng X., Zhao M., Sun T., Fan Z. (2021). Health risk assessment of heavy metal(loid)s in park soils of the largest megacity in China by using Monte Carlo simulation coupled with Positive matrix factorization model. J. Hazard. Mater..

[9] Sanaei F., Amin M.M., Alavijeh Z.P., Esfahani R.A., Sadeghi M., Bandarrig N.S., Fatehizadeh A., Taheri E., Re-zakazemi M. (2021). Health risk assessment of potentially toxic elements intake via food crops consumption: Monte Carlo simu-lation-based probabilistic and heavy metal pollution index. Environ. Sci. Pollut. Res..

[10] Dai H., Jing S., Wang H., Ma Y., Li L., Song W., Kan H. (2017). VOC characteristics and inhalation health risks in newly renovated residences in Shanghai, China. Sci. Total Environ..

[11] Huang D., Liu M., Zhang J., Wang Y. Research on risk assessment based on Monte Carlo simulation and dose-response multistage model. Proceedings of the 2010 3rd International Conference on Biomedical Engineering and Informatics.

[12] Simon S.L., Hoffman F.O., Hofer E. (2015). The two-dimensional Monte Carlo: A new methodologic paradigm for dose reconstruction for epidemiological studies. Radiat. Res..

[13] Wang C.-C., Niu Z.-G., Zhang Y. (2013). Health risk assessment of inhalation exposure of irrigation workers and the public to trihalomethanes from reclaimed water in landscape irrigation in Tianjin, North China. J. Hazard. Mater..

[14] Luo K., Zeng Y., Li M., Man Y., Zeng L., Zhang Q., Luo J., Kang Y. (2021). Inhalation bioacessibility and absorption of polycyclic aromatic hydrocarbons (PAHs) in indoor PM2.5 and its implication in risk assessment. Sci. Total Environ..

[15] Qin N., He W., Liu W., Kong X., Xu F., Giesy J.P. (2020). Tissue distribution, bioaccumulation, and carcinogenic risk of polycyclic aromatic hydrocarbons in aquatic organisms from Lake Chaohu, China. Sci. Total Environ..

[16] Wang J., Odinga E.S., Zhang W., Zhou X., Yang B., Waigi M.G., Gao Y. (2019). Polyaromatic hydrocarbons in biochars and human health risks of food crops grown in biochar-amended soils: A synthesis study. Environ. Int..

[17] Wang B., Jin L., Ren A., Yuan Y., Liu J., Li Z., Zhang L., Yi D., Wang L.-L., Zhang Y. (2015). Levels of Polycyclic Aromatic Hydrocarbons in Maternal Serum and Risk of Neural Tube Defects in Offspring. Environ. Sci. Technol..

[18] Han J., Liang Y., Zhao B., Wang Y., Xing F., Qin L. (2019). Polycyclic aromatic hydrocarbon (PAHs) geographical distribu-tion in China and their source, risk assessment analysis. Environ. Pollut..

[19] Rogge W.F., Hildemann L.M., Mazurek M.A., Cass G.R., Simoneit B.R.T. (1993). Sources of fine organic aerosol. 3. Road dust, tire debris, and organometallic brake lining dust: Roads as sources and sinks. Environ. Sci. Technol..

[20] Da Silva F.C., Felipe M., de Castro D.E.F., Araujo S.C.D., Sisenando H.C.N., de Medeiros S.R.B. (2021). A look beyond the priority: A systematic review of the genotoxic, mutagenic, and carcinogenic endpoints of non-priority PAHs *. Environ. Pollut..

[21] Qin N., Kong X.-Z., He W., He Q.-S., Liu W.-X., Xu F.-L. (2021). Dustfall-bound polycyclic aromatic hydrocarbons (PAHs) over the fifth largest Chinese lake: Residual levels, source apportionment, and correlations with suspended particulate matter (SPM)-bound PAHs in water. Environ. Sci. Pollut. Res..

[22] Boffetta P., Jourenkova N., Gustavsson P. (1997). Cancer risk from occupational and environmental exposure to polycyclic aromatic hydrocarbons. Cancer Causes Control..

[23] Kim K.-H., Jahan S.A., Kabir E., Brown R.J.C. (2013). A review of airborne polycyclic aromatic hydrocarbons (PAHs) and their human health effects. Environ. Int..

[24] Kuang D., Zhang W., Deng Q., Zhang X., Huang K., Guan L., Hu D., Wu T., Guo H. (2013). Dose-Response Relationships of Polycyclic Aromatic Hydrocarbons Exposure and Oxidative Damage to DNA and Lipid in Coke Oven Workers. Environ. Sci. Technol..

[25] Miller B.G., Doust E., Cherrie J.W., Hurley J.F. (2013). Lung cancer mortality and exposure to polycyclic aromatic hydrocar-bons in British coke oven workers. BMC Public Health.

[26] Zhu Y., Duan X., Qin N., Li J., Tian J., Zhong Y., Chen L., Fan R., Yu Y., Wu G. (2019). Internal biomarkers and external estimation of exposure to polycyclic aromatic hydrocarbons and their relationships with cancer mortality in a high cancer incidence area. Sci. Total Environ..

[27] Zhen Z., Yin Y., Chen K., Zhen X., Zhang X., Jiang H., Wang H., Kuang X., Cui Y., Dai M. (2021). Concentration and atmospheric transport of PM2.5-bound polycyclic aromatic hydrocarbons at Mount Tai, China. Sci. Total Environ..

[28] Zhou S., Hwang B., Lakey P., Zuend A., Abbatt J.P.D., Shiraiwa M. (2019). Multiphase reactivity of polycyclic aromatic hydrocarbons is driven by phase separation and diffusion limitations. Proc. Natl. Acad. Sci. USA.

[29] Petit P., Maître A., Persoons R., Bicout D.J. (2019). Lung cancer risk assessment for workers exposed to polycyclic aromatic hydrocarbons in various industries. Environ. Int..

[30] Li R., Cheng M., Cui Y., He Q., Guo X., Chen L., Wang X. (2020). Distribution of the Soil PAHs and Health Risk Influenced by Coal Usage Processes in Taiyuan City, Northern China. Int. J. Environ. Res. Public Health.

[31] Zhang M., Xie J., Wang Z., Zhao L., Zhang H., Li M. (2016). Determination and source identification of priority polycyclic aromatic hydrocarbons in PM2.5 in Taiyuan, China. Atmos. Res..

[32] Yan Y., He Q., Guo L., Li H., Zhang H., Shao M., Wang Y. (2017). Source apportionment and toxicity of atmospheric poly-cyclic aromatic hydrocarbons by PMF: Quantifying the influence of coal usage in Taiyuan, China. Atmos. Res..

[33] Liu Y.W., Qin N., Liang W.G., Chen X., Hou R., Kang Y.J., Guo Q., Cao S.Z., Duan X.L. (2020). Polycycl. Aromatic Hydrocarbon Exposure of Children in Typical Household Coal Combustion Environments: Seasonal Variations, Sources, and Carcinogenic Risks. Int. J. Environ. Res. Public Health.

[34] China Statistical Publishing House (2009). Shanxi Statistical Yearbook.

[35] Shanxi Province Bureau of Statistics (2012). Tabulation on the 2010 Population Census of Shanxi Province.

[36] Xia Z., Duan X., Tao S., Qiu W., Liu D., Wang Y., Wei S., Wang B., Jiang Q., Lu B. (2013). Pollution level, inhalation exposure and lung cancer risk of ambient atmospheric polycyclic aromatic hydrocarbons (PAHs) in Taiyuan, China. Environ. Pollut..

[37] Duan X., Wang B., Zhao X., Shen G., Xia Z., Huang N., Jiang Q., Lu B., Xu D., Fang J. (2014). Personal inhalation exposure to polycyclic aromatic hydrocarbons in urban and rural residents in a typical northern city in China. Indoor Air.

[38] USEPA (2019). Guidelines for human exposure assessment. Risk Assessment Forum.

[39] Chen S.-C., Liao C.-M. (2006). Health risk assessment on human exposed to environmental polycyclic aromatic hydrocarbons pollution sources. Sci. Total Environ..

[40] Liao C.M., Chio C.P., Chen W.Y., Ju Y.R., Li W.H., Cheng Y.H., Liao V.H.C., Chen S.C., Ling M.P. (2011). Lung cancer risk in relation to traffic-related nano/ultrafine particle-bound PAHs exposure: A preliminary probabilistic assessment. J. Hazard. Mater..

[41] Zhao X., Duan X. (2014). Exposure Factors Handbook of Chinese Population.

[42] Culle A., Frey H. (1999). Probabilistic Techniques in Exposure Assessment: A Handbook for Dealing with Variability and Uncertainty in Models and Inputs.

[43] Zhang M., Wang J., Zhao Q., Mishra V., Fan J., Sun Y. (2020). Polycyclic aromatic hydrocarbons (PAHs) and esophageal carcinoma in Handan-Xingtai district, North China: A preliminary study based on cancer risk assessment. Environ. Monit. Assess..

[44] Huang Y., Wang J., Fu N., Zhang S., Du W., Chen Y., Wang Z., Qi M., Wang W., Zhong Q. (2021). Inhalation exposure to size-segregated fine particles and particulate PAHs for the population burning biomass fuels in the Eastern Tibetan Plateau area. Ecotoxicol. Environ. Saf..

[45] Bi C., Chen Y., Zhao Z., Li Q., Zhou Q., Ye Z., Ge X. (2020). Characteristics, sources and health risks of toxic species (PCDD/Fs, PAHs and heavy metals) in PM2.5 during fall and winter in an industrial area. Chemosphere.

[46] Qin N., He W., Kong X.Z., Liu W.X., He Q.S., Yang B., Ouyang H.L., Wang Q.M., Xu F.L. (2013). Atmospheric parti-tioning and the air-water exchange of polycyclic aromatic hydrocarbons in a large shallow Chinese lake (Lake Chaohu). Chemosphere.

[47] Zhu Y., Duan X., Qin N., Lv J., Wu G., Wei F. (2019). Health risk from dietary exposure to polycyclic aromatic hydrocarbons (PAHs) in a typical high cancer incidence area in southwest China. Sci. Total Environ..

[48] Hoseini M., Yunesian M., Nabizadeh R., Yaghmaeian K., Ahmadkhaniha R., Rastkari N., Parmy S., Faridi S., Rafiee A., Naddafi K. (2016). Characterization and risk assessment of polycyclic aromatic hydrocarbons (PAHs) in urban atmospheric Particulate of Tehran, Iran. Environ. Sci. Pollut. Res..

[49] Nisbet I.C.T., Lagoy P.K. (1992). Toxic equaivalency factors (TEFs) for Polycyclic Aromatic-Hydrocarbons (PAHs). Regul. Toxicol. Pharmacol..

[50] Layton D.W. (1993). Metabolically consistent breathing rates for use in dose assessments. Health Phys..

[51] USEPA (2011). Exposure Factors Handbook.

