# Supplementary material for: Key Factors for Improving the Carcinogenic Risk Assessment of PAH Inhalation Exposure by Monte Carlo Simulation
Source: Int J Environ Res Public Health. 2021 Oct 22;18(21):11106. doi: 10.3390/ijerph182111106 (PMC8583189; doi:10.3390/ijerph182111106)
Supplement: Supplementary file 1 [file ijerph-18-11106-s001.zip › ijerph-1358479-supplementary.pdf]

This supporting information provides tables, figures and text addressing

Table S1 Toxicity equivalency factors (TEFs) and reference dose for the PAHs (Nisbet and Lagoy 1992)

Table S2 Estimation of BMR (Basal Metabolism Rate) of population in Taiyuan

Figure S1. Comparison of age structure difference between participants and local population

Figure S2. Correlation between IR and BW in participants

Figure S3 Relationship between inhalation rate and bodyweight by traditional Monte Carlo simulation

Text S1: Estimation of inhalation rate

Table S1. Toxicity equivalency factors (TEFs) and reference dose for the PAHs [49].

Compound	TEFs	Compound	TEFs
Nap	0.001	BaA	0.1
Acy	0.001	Chr	0.01
Ace	0.001	BbF	0.1
Flo	0.001	BkF	0.1
Phe	0.001	BaP	1
Ant	0.01	IcdP	0.1
Fla	0.001	DahA	1
Pyr	0.001	BghiP	0.01

Table S2. Estimation of BMR (basal metabolic rate) of population in Taiyuan.

Age Group (Years)	Male	Female
<18	370 + 20H + 52W − 25A	1873 + 13H + 39W − 18A
18–30	63W + 2896	62W + 2036
30–60	48W + 3653	34W + 3538
>60	370 + 20H + 52W − 25A	1873 + 13H + 39W − 18A

H is the body height, W is the body weight, A is age.

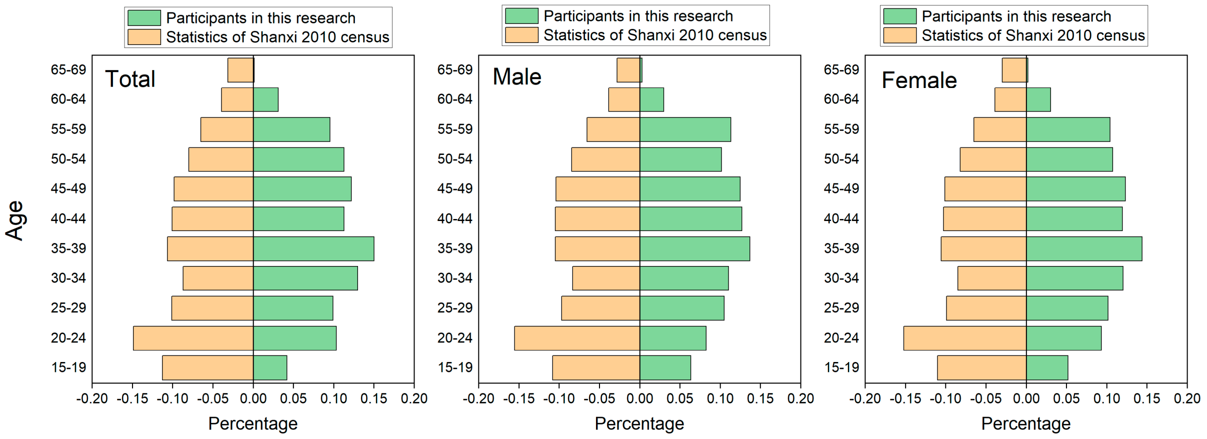

Figure S1. Comparison of age structure difference between participants and local population.

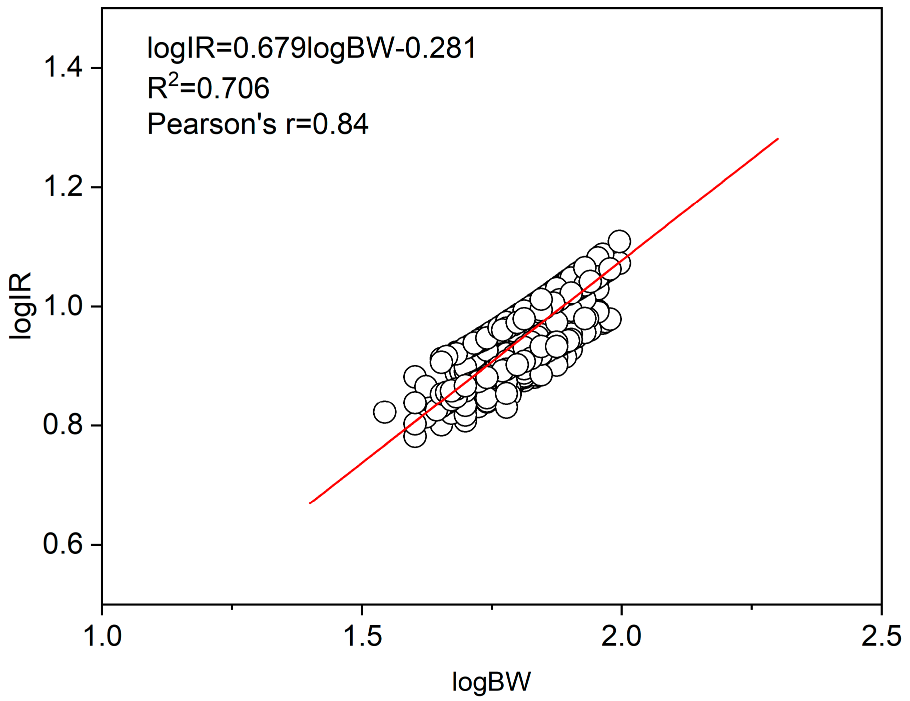

Figure S2. Correlation between IR and BW in participants.

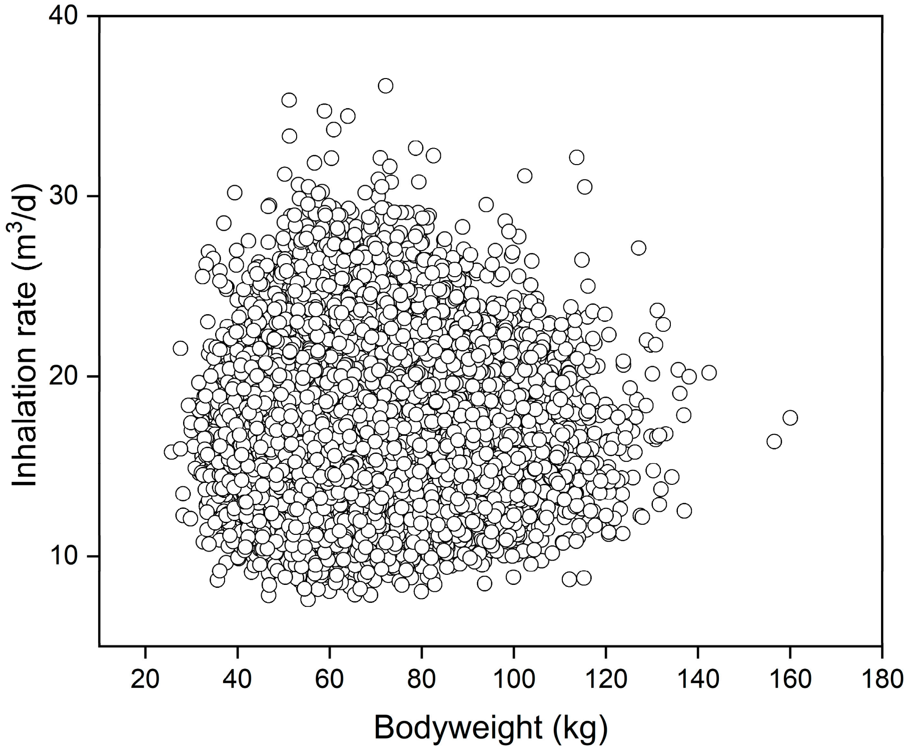

Figure S3. Relationship between inhalation rate and body weight by traditional Monte Carlo simulation.

Text S1. Estimation of inhalation rate.

Subject inhalation rates were estimated based on the basal metabolic rate (BMR):

$$IR = \frac{BMR \times MET \times U \times VQ}{1440} \quad \text{(S1)}$$

where BMR [50] was defined as the minimum amount of energy required to support basic cellular respiration while at rest and not actively digesting food (kJ/day), IR is inhalation rate (L/min), U is oxygen uptake per unit energy (0.05 L/kJ), VQ is ventilation equivalent (27 unitless), MET (metabolic equivalent) is the coefficient associated with physical activity. MET = 1.5 was applied in this study [51].
